# Different Topological Properties of EEG-Derived Networks Describe Working Memory Phases as Revealed by Graph Theoretical Analysis

**DOI:** 10.3389/fnhum.2017.00637

**Published:** 2018-01-12

**Authors:** Jlenia Toppi, Laura Astolfi, Monica Risetti, Alessandra Anzolin, Silvia E. Kober, Guilherme Wood, Donatella Mattia

**Affiliations:** ^1^Department of Computer, Control and Management Engineering, Sapienza University of Rome, Rome, Italy; ^2^Neuroelectrical Imaging and Brain-Computer Interface Laboratory, Fondazione Santa Lucia IRCCS, Rome, Italy; ^3^Department of Psychology, University of Graz, Graz, Austria; ^4^BioTechMed-Graz, Graz, Austria

**Keywords:** EEG, brain networks, working memory, sternberg task, connectivity, graph theory

## Abstract

Several non-invasive imaging methods have contributed to shed light on the brain mechanisms underlying working memory (WM). The aim of the present study was to depict the topology of the relevant EEG-derived brain networks associated to distinct operations of WM function elicited by the Sternberg Item Recognition Task (SIRT) such as encoding, storage, and retrieval in healthy, middle age (46 ± 5 years) adults. High density EEG recordings were performed in 17 participants whilst attending a visual SIRT. Neural correlates of WM were assessed by means of a combination of EEG signal processing methods (i.e., time-varying connectivity estimation and graph theory), in order to extract synthetic descriptors of the complex networks underlying the encoding, storage, and retrieval phases of WM construct. The group analysis revealed that the encoding phase exhibited a significantly higher *small-world* topology of EEG networks with respect to storage and retrieval in all EEG frequency oscillations, thus indicating that during the encoding of items the global network organization could “*optimally*” promote the information flow between WM sub-networks. We also found that the magnitude of such configuration could predict subject behavioral performance when memory load increases as indicated by the negative correlation between Reaction Time and the *local efficiency* values estimated during the encoding in the alpha band in both 4 and 6 digits conditions. At the local scale, the values of the *degree index* which measures the degree of in- and out- information flow between scalp areas were found to specifically distinguish the *hubs* within the relevant sub-networks associated to each of the three different WM phases, according to the different role of the sub-network of regions in the different WM phases. Our findings indicate that the use of EEG-derived connectivity measures and their related topological indices might offer a reliable and yet affordable approach to monitor WM components and thus theoretically support the clinical assessment of cognitive functions in presence of WM decline/impairment, as it occurs after stroke.

## Introduction

The working memory (WM) is a non-unitary construct that involves the temporary maintenance and manipulation of information either recently acquired or retrieved from long-term storage (Baddeley, 1996). The Baddeley's model is one of the most recognized among the several current models describing the operating principles of WM (D'Esposito and Postle, 2015). It encompasses diverse separable but interacting subsystems such as: 2 unimodal storage sub-systems (phonological loop for verbal material and visuo-spatial sketchpad for visuo-spatial material), a flexible system (central executive) which is responsible for the control and regulation of the storage sub-systems and a multimodal system with limited capacity storage (episodic buffer) that allows the interaction between the various components of WM and the interface with long-term memory (LTM) (Baddeley, 2000, 2010).

The Sternberg Item Recognition Task (SIRT; Sternberg, 1966) has been largely used in cognitive neuroscience to assess WM capacity in terms of storage and data retrieval (Nosofsky et al., 2011; Corbin and Marquer, 2013). It allows for a segregation of encoding, executive maintenance and retrieval processes (not manipulation) regarded as central within the multi-component model of WM. The SIRT is also relatively free from practice effects (Kristofferson, 1972). The SIRT was initially introduced to investigate the neurophysiological processes at the basis of WM by means of indirect behavioral measures (Sternberg, 1966, 1969). Its application was extended later into the field of neuroimaging techniques, functional magnetic resonance imaging (fMRI), electroencephalography (EEG), and magnetoencephalography (MEG) aiming at directly measuring the neural correlates underpinning WM processes (Rypma et al., 1999; Cairo et al., 2004; Payne and Kounios, 2009; Keren-Happuch et al., 2012).

In this regard, several fMRI studies have shown that verbal WM processing in adult humans requires the involvement of a large network of areas which includes bilateral dorso-lateral, prefrontal, left inferior frontal, middle and superior frontal areas, premotor and supplementary motor areas as well as inferior parietal and superior temporal areas, the insula and parts of the cerebellum (Smith and Jonides, 1998; Marvel and Desmond, 2010; Luis et al., 2015). Further studies using SIRT found specific patterns of activation for each of the three phases of WM (encoding, storage, and retrieval) which were also sensitive to WM load levels (Rypma and D'Esposito, 1999; Cairo et al., 2004; Chen and Desmond, 2005; Marvel and Desmond, 2010; Thürling et al., 2012; Vergauwe et al., 2015).

Evidence for specific brain oscillatory responses elicited during the different phases of WM emerged from EEG and MEG studies using the SIRT. In particular, the maintenance (storage) phase of verbal SIRT was associated with oscillatory power in theta (4–8 Hz) predominantly over the frontal midline and left temporal-parietal sites (Payne and Kounios, 2009; Brookes et al., 2011; Kottlow et al., 2015) as well as in alpha (8–13 Hz) power over the parietal midline, the parieto-occipital and left tempo-parietal regions (Jensen et al., 2002; Scheeringa et al., 2009; Heinrichs-Graham and Wilson, 2015; Xie et al., 2016). Changes in EEG power spectra such as an increase of bilateral frontal delta, frontal-midline theta, and temporo-parietal alpha and a decrease of beta and gamma activities in frontal and occipital areas have been observed as function of WM load (Jensen and Tesche, 2002; Hwang et al., 2005; Payne and Kounios, 2009; Axmacher et al., 2010; Brookes et al., 2011; Roux and Uhlhaas, 2014; Zakrzewska and Brzezicka, 2014; Maurer et al., 2015; Gurariy et al., 2016). Delta power also varies as a function of stimulus type (so-called old-new effect; Kayser et al., 2007; Mathes et al., 2012).

To fully understand brain functions, functional neuroimaging methods have been also applied to investigate the dynamics within networks of brain areas that underlie specific cognitive processes (such as WM), and how a brain damage-induced disruption of neural circuits could account for behavioral impairments (Honey and Sporns, 2008; Cramer et al., 2011; Grefkes and Fink, 2011, 2014). In this regard, functional connectivity estimation was applied to track age-related changes in brain connectivity in a group of children and adolescents performing a modified version of the SIRT (van den Bosch et al., 2014). Task-related networks were identified for encoding (including left motor area, right prefrontal, parietal, and occipital cortex cerebellum) and recognition (including anterior and posterior cingulate cortex, right motor area, cerebellum, left parietal, and prefrontal cortex) phases and their load-induced modulation also correlated with age (Woodward et al., 2013; van den Bosch et al., 2014).

In this study, we take advantage of high temporal resolution of EEG technique and its relatively low-cost and easiness to use to isolate salient descriptors of WM processes as elicited by SIRT, in healthy middle age condition. To this purpose, a combined approach based on EEG-derived connectivity patterns and graph theory (Baccalá and Sameshima, 2001; Milde et al., 2010; Rubinov and Sporns, 2010; Astolfi et al., 2013) was adopted. We expected such combined approach to return quantitative measurements specific for the three different WM phases (encoding, storage and retrieval) and sensitive to different memory workload. The relationship between extracted neurophysiological indices and subject memory performance was also assessed to explore to what extent the estimated EEG networks topology would account for memory behavior. Here, we targeted middle-age population (i.e., between 40 and 50 years) to limit possible confounding effects on the stability of EEG network measures as due to changes in memory task-related neural activity that may emerge from middle age (i.e., fourth decade) onward (Aine et al., 2006, 2011; Grady et al., 2006; Mattay et al., 2006; MacPherson et al., 2014).

The ultimate goal is to provide affordable (EEG-based) computational instruments to measure the electrophysiological dynamics at the level of brain networks that underpin theoretical models of WM processes. As such, this EEG-based network approach could also serve as an objective counterpart of the behavioral assessment of WM impairments which often occur in acquired brain lesions (e.g., stroke) and to ground future cognitive rehabilitative strategy design (Kober et al., 2015, 2017).

## Materials and methods

### Participants

Seventeen healthy subjects (age: 46 ± 5 years old; 6 males; education: 14.8 ± 3 years; see Table 1) were enrolled in the study. All participants except one were right-handed with normal or corrected-to-normal vision. No participant reported a history of neurological or psychiatric diseases; in addition, they were all screened for intake medications and none was receiving any pharmacological treatment affecting cognitive functions. Participants underwent some subtests (Similarities, Information, Coding, Picture Completion, Mosaic Test) from the German adaptation of the Wechsler Adult Intelligence Scale (WAIS III, Von Aster et al., 2006), for a general screening of the cognitive functions and also a deep evaluation of the memory functions. In particular, for the evaluation of the verbal and visuo-spatial memory, subjects performed the Corsi Block Tapping Test (CBTT) (Corsi and Michael, 1972), the Visual and Verbal Memory Test (VVM 2) (Schellig and Schächtele, 2009), the Digit Span (Härting, 2000), the Verbal Learning Memory Test (VLMT), the Nonverbal Learning Test (NVLT), the Verbal Learning Test (VLT) (Sturm and Willmes, 1999). All the subjects obtained normal scores in all the investigated cognitive domains (Table 1). This study was carried out in accordance with the recommendations provided in the declaration of Helsinki. All participants provided written informed consent according to the convention of Helsinki. The ethics committee of the University of Graz approved the study. All participants received monetary reward for their participation to the study.

**Table 1:** Table reporting the demographical data of the participants and the results of the cognitive screening.

**Code**		**1**	**2**	**3**	**4**	**5**	**6**	**7**	**8**	**9**	**10**	**11**	**12**	**13**	**14**	**15**	**16**	**17**
Gender		F	M	F	M	F	F	M	F	F	F	M	F	M	M	F	F	F
Age		43	41	49	47	49	43	40	43	44	63	45	56	45	49	42	40	51
Handedness		R	R	R	R	R	R	R	R	R	R	L	R	R	R	R	R	R
Highest education		U	HS	HS	HS	CS	U	U	U	HS	CS	U	HS	U	HS	HS	HS	HS
Cognitive tests	CBTT forward task	10	8	8	8	8	8	8	8	6	8	5	7	10	11	11	6	8
	Digit span forward task	11	12	10	8	11	9	10	13	9	10	10	11	9	13	9	12	11
	VVM2 – City map 1	40	51	49	59	34	51	47	42	47	65	51	43	49	51	51	64	56
	VVM2 – Construction 1	34	55	44	57	41	55	48	46	50	71	50	55	57	60	60	46	58
	CBTT backwards task	6	7	6	6	7	5	7	6	6	6	8	5	8	8	10	7	7
	Digit span backwards task	7	10	7	7	10	7	8	10	10	7	10	10	7	10	9	13	8
	VLT	61	54	43	30	32	34	48	50	46	56	52	57	31	61	63	57	46
	NVLT	46	37	41	47	40	44	43	45	45	43	38	37	37	38	44	58	41
	VLMT T—ΣDg1–5	60	63	51	63	60	63	54	48	67	58	54	60	33	63	67	67	63
	VLMT T—Dg7	52	62	33	62	41	56	44	48	60	52	41	61	33	56	62	52	56
	VLMT T—Dg5-Dg7	40	58	33	58	45	52	45	45	58	49	34	58	40	51	58	45	55
	VLMT T—W-F	54	54	48	54	53	54	53	48	54	53	45	62	33	54	54	48	48
	VVM2—City map 2	54	48	32	59	28	41	41	49	41	72	49	58	41	48	54	60	62
	VVM2—Construction 2	32	54	42	54	35	47	38	47	54	71	56	51	62	56	66	42	48
	WAIS-IV Similarities	57	47	50	63	67	47	57	47	53	60	57	60	57	57	60	57	53
	WAIS-IV Picture completion	50	50	67	63	50	43	50	40	57	53	67	50	57	57	67	50	63
	WAIS-IV Mosaic test/block design	67	60	67	63	53	43	63	40	47	50	60	53	70	50	63	63	60
	WAIS-IV Number-symbol test	40	43	40	67	50	47	50	33	40	70	50	47	73	57	57	40	73
	WAIS-IV Information	57	57	70	57	60	57	60	50	53	63	63	47	63	50	60		60

*F, female; M, male; R, right; L, left; U, university; HS, high school; CS, compulsory school*.

### Data acquisition and experimental paradigm

The EEG potentials were recorded from 60 scalp electrodes embedded in a lycra cap, with a left mastoid reference and a ground at Fpz. Horizontal and vertical electro-oculogram (EOG) signals were recorded from 3 electrodes in total, two placed on the outer canthi of the eyes and one below the right eye, respectively. EEG signals were amplified (BrainAmp; Brain Products GmbH, Munich, Germany) and filtered by means of a [0.01–100] Hz band-pass filter prior to digitization at 500 Hz. Electrode impedances were kept below 5 and 10 kOhms for the EEG and EOG recording, respectively.

**Figure 1:**
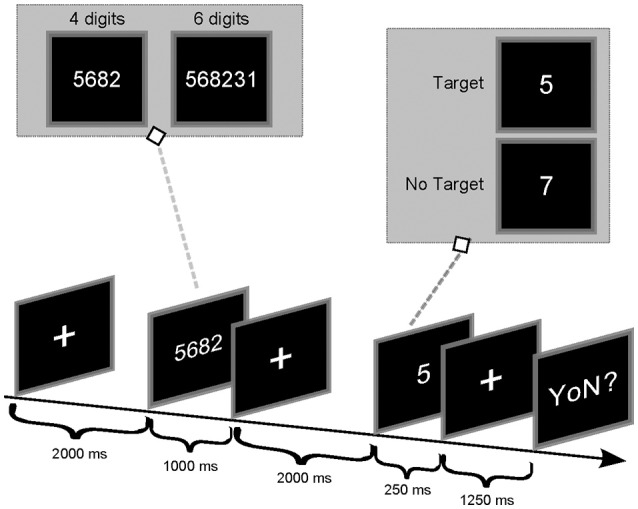
Timing of the Sternberg experiment.

After 2 min of resting EEG acquisition (of eyes open and close), each subject performed the Sternberg task (Sternberg, 1966). Accordingly, the experimental procedure to deliver the paradigm was as follows (Figure 1): first, a series of digits was visually presented to the participants who had to memorize it (encoding phase); then, the participants had to retain the memorized information for a fixed period (storage phase) and finally, participants had to retrieve such memorized content in a brief time interval (retrieval phase). In particular, participants were asked to remember a set of unique digits (between 1 and 9), and then a probe stimulus in the form of a digit was presented. Subjects were instructed to answer, as quickly as possible, whether the probe was in the previously presented set of digits or not. The size of the initial set of digits determined the WM load required to the subject to execute the task (4 digits → easy, low workload; 6 digits → difficult, high workload). Each trial started with a 2 s presentation of a fixation cross in the middle of the screen. Afterwards, a “memory set” of four (e.g., 5682) or 6 digits (e.g., 146372) was presented (1 s) to allow for memorization (encoding phase). The presentation of the digit series was then followed by a fixation cross, displayed for 2 s (storage period). A single probe digit was then presented for 250 ms (retrieval phase) followed by a fixation cross presented for 1,250 ms. Afterwards, the question “yes or no?” appeared on the screen (maximum duration of 1,500 ms) and the participant had to answer: if the probe was a member of the preceding digit series, the participant had to press the left button (Target Condition) whereas, the right button had to be pressed in case the probe was not part of the series (NoTarget Condition). A new trial would start upon participant answer. The probability of Target condition (36 trials in total) was 0.5 and the digits contained in each trial were presented in a randomized order. The conditions 4/6 digits and Target/NoTarget were also randomized along the recording session.

### Behavioral data

We collected reaction times (RT) and the percentage of correct answers for each subject and each condition (Target/NoTarget and 4–6 digits). To examine any effect of task-related complexity and task-related trials on the subject behavioral performances, 2 separate two-way repeated measures ANOVAs with digits number (DIGITS; 4 or 6) and target type (TYPE; Target/NoTarget) as within main factors were performed considering the percentage of correct answers and reaction times (RTs) parameters as dependent variables.

### Data pre-processing

EEG signals were downsampled to 100 Hz (with anti-aliasing low-pass filter) to optimize the following connectivity analysis and then band-pass filtered in the range (1–45) Hz in order to isolate the EEG spectral content of interest. Independent Component Analysis (ICA—fastICA algorithm) was used to remove ocular artifacts (i.e., the blinks-related IC was removed on the basis of its temporal content and spatial distribution—mainly located over frontal scalp areas). EEG traces were segmented in relation with the specific timing of the paradigm, (0–4,500) ms (period of interest) according to the onset of the first screen containing the digits series and classified according to different conditions (Target_4digits, NoTarget_4digits, Target_6digits, NoTarget_6digits). Only trials correctly executed were included in the analysis. Residual artifacts were then removed by means of a semi-automatic procedure based on a threshold criterion (±80 μV). Only the artifacts-free trials were used for further analysis (no less than 30 trials per condition were considered for final analysis). The entire pre-processing procedure was performed by means of Brain Vision Analyzer 1.0 software (Brain Products GmbH).

### Time-varying connectivity estimation

Pre-processed EEG signals were subjected to a time-varying connectivity estimation process for each subject and each experimental condition (4/6 digits, Target/NoTarget). Several time-domain or frequency-domain measures were developed to estimate connectivity from EEG data in terms of correlation/coherence, statistical dependencies, or causal interaction among data series. Some of these measures are deemed to be less prone to volume conduction effects (Haufe et al., 2013), such as imaginary coherence (Nolte et al., 2004) or phase slope index (Nolte et al., 2008) but return non-causal, undirected measures, and are based on a pairwise approach, which results in high rates of false positives when the network complexity increases (Kus et al., 2004). Here, we employed a time-varying adaptation of Partial Directed Coherence (PDC), a spectral multivariate estimator which provides with the directed influences between any given pair of signals in a multivariate data set (Baccalá and Sameshima, 2001; Astolfi et al., 2006; Toppi et al., 2016b). Such time-varying adaptation is based on the General Linear Kalman Filter (GLKF) (Milde et al., 2010) which is able to follow temporal dynamics of brain networks with high temporal resolution in high density EEG data (Toppi et al., 2016a). We used it to estimate the relationships between signals for all frequency samples in the range (1–45) Hz and for all the samples in the time interval (0–4,500) ms.

The connectivity patterns contrasted with the baseline period were estimated for each time sample and averaged in the five frequency bands-of-interest and in three time intervals (periods-of-interest). The frequency bands were individually defined according to the Individual Alpha Frequency (IAF-10 ± 0.9 Hz), as determined by means of the Fast Fourier Transform spectra of 2 min resting EEG (recorded before SIRT execution) over posterior leads (parietal, parieto-occipital, and occipital). The following frequency bands were then considered: Delta (IAF-8/IAF-6), Theta (IAF-6/IAF-2), Alpha (IAF-2/IAF+2), Beta (IAF+2/IAF+14) and Gamma (IAF+15/IAF+30; Klimesch, 1999). The three periods-of-interest correspond to: (0–1,000) ms (encoding phase); (1,000–3,000) ms (storage phase) and (3,000–4,500) ms (retrieval phase). The analysis was conducted only on Target condition.

Any relevant changes in the time-varying connectivity matrices related to the different experimental conditions were evaluated by means of statistical comparisons (independent samples *t*-test) performed for each task condition (Target_4digits, NoTarget_4digits, Target_6digits, NoTarget_6digits) between each post-stimulus time window (encoding, storage, retrieval) and the baseline period. The baseline period was the time interval (-1000-0) ms preceding the appearance of the digits series (subjects fixing a cross on the screen). Time samples were used as observations for statistical test. The test was repeated for each frequency band and each subject. The significance level was set at 5%. A False Discovery Rate (FDR) was conducted for multiple comparison correction (Benjamini and Yekutieli, 2001).

The analysis pipeline was performed in Matlab environment (MATLAB, 2011).

### Graph indices

The main global and local properties of the estimated networks were quantified by means of indices derived from the graph theory. Such indices are defined on the basis of a binary adjacency matrix *G*, obtained by comparing each entry of the connectivity matrix A with its corresponding threshold as follows:

(1) $$G_{ij}(f,t)=\{\begin{array}{c} 1\to A_{ij}(f,t)\geq \tau_{ij}(f,t)\\ 0\to A_{ij}(f,t)<\tau_{ij}(f,t) \end{array}$$

where *G*_*ij*_ and *A*_*ij*_ represent the entry *(i,j)* of the adjacency matrix G and the PDC matrix A, respectively, and τ_*ij*_ is the corresponding threshold. As mentioned above, we adopted a statistical approach for the threshold definition in order to avoid the attribution of false properties to the networks due to the application of an empirical thresholds (Toppi et al., 2012). Accordingly, the threshold τ_ij_ corresponds to the 95th percentile (corrected for multiple comparisons by FDR) of the PDC distribution obtained for the baseline condition.

The estimated adjacency matrices were then, used to extract local and global indices as described below. To avoid network-size effects, each global index was normalized by its corresponding value obtained from 100 random graphs generated by fixing the connection density of the original network. Random graphs were thus used, as reference level for the description of global properties of WM networks (see below).

Local and global indices were computed by means of routines provided in Brain Connectivity Toolbox developed for Matlab environment (Rubinov and Sporns, 2010).

#### General properties of the network

The human brain can be viewed as a large-scale complex network that is simultaneously segregated and integrated via specific connectivity patterns (Bullmore and Sporns, 2009). We selected the three indices—local and global efficiency and small-worldness—that are widely utilized to describe the general topological properties of a network, thus reflecting the integration and segregation of the information flow between areas (Sporns, 2013b).

##### Global efficiency (GE)

The GE is a global measure (considering all the connections in the whole-network) of how efficiently a network exchanges information internally. It is defined as the average of the inverse of the geodesic distance (shortest path between two nodes in the network) and it represents the efficiency of the communication between all the nodes within the network (Latora and Marchiori, 2001). It can be defined as follows:

(2) $$GE=\frac{1}{N(N-1)}\sum_{i\neq j}\frac{1}{d_{ij}}$$

where *N* represents the number of nodes in the graph and *d*_*ij*_ the geodesic distance between *i* and *j*.

##### Local efficiency (LE)

The LE is a measure of the fault tolerance of a network. It verifies whether the communication between nodes is still efficient when a node is removed from the network. The higher the LE, the greater the robustness of the network at local scale.

The LE is the average of the global efficiencies computed on each sub-graph *S*_*i*_ belonging to the network and it represents the efficiency of the communication between all the nodes around the node *i* in the network (Latora and Marchiori, 2001). It can be defined as follows:

(3) $$LE=\frac{1}{N}\sum_{i=1}^{N}E_{g}(S_{i})$$

where *N* represents the number of nodes in the graph and *S*_*i*_ the sub-graph obtained deleting the *i*th row and the *i*th column from the original adjacency matrix.

##### Small-worldness (SW)

It has been suggested that the human networks are organized to optimize efficiency, due to a small-world topology allowing simultaneous global and local parallel information processing (Bassett and Bullmore, 2006). SW is a measure of a network global organization in terms of its integration and segregation properties. Small-world topology is typical of networks highly segregated (nodes organized according to clusters) and highly integrated (high communication speed between electrodes).

A network *G* is defined as small-world network if *L*_*G*_ ≥ *L*_*rand*_ and *C*_*G*_ ≫ *C*_*rand*_ where *L*_*G*_ and *C*_*G*_ represent the characteristic path length (Sporns et al., 2004) and the clustering coefficient (Fagiolo, 2007) of a generic graph and *L*_*rand*_ and C_*rand*_ represent the correspondent quantities for a random graph (Watts and Strogatz, 1998). On the basis of this definition, a measure of small-worldness of a network can be introduced as follows:

(4) SW=CGCrandLGLrand

being a *small-world* network if S > 1 (Humphries and Gurney, 2008).

#### Local properties of the network

The topology of the networks was further investigated by computing the degree index for each scalp electrode to characterize the (local) level of in- and out- information flows exchanged within the network.

##### Degree

The degree of a node is the number of connections involving it. As such the degree is the simplest index identifying *hubs* in graphs. In directed networks, the indegree is the number of inward links and the outdegree is the number of outward links (Sporns et al., 2004). It can be defined as follows:

(5) $$k_{f}=\sum_{j\in N,j\neq f}g_{fj}+\sum_{i\in N,i\neq f}g_{if}$$

where *k*_*f*_ is the degree of node *f* and *g*_*ij*_ represents the entry *ij* of the adjacency matrix *G*. The degree of a specific electrode was normalized with respect to the network density, in order to capture local changes and not a general increase/decrease of the network density.

All the extracted global and local indices were subjected to a two-way ANOVA with memory phases (PHASES: Encoding, Storage, Retrieval) and digits number (DIGITS: 4, 6) as main *within-subject* factors. Duncan's *post-hoc* test was used to verify differences between the ANOVA levels. FDR was further applied to correct for multiple ANOVAs. Furthermore, to explore the relationship between the indices extracted for each memory phase and the relative behavioral data (correct answers rate, reaction time) a Pearson correlation analysis was performed. FDR was applied to reduce type I errors due to multiple correlations.

## Results

### Behavioral results

The overall behavioral data obtained from each subject is reported in Table 2. All the participants showed a percentage of correct answers above 80% (except for subject 5 in 6 Digits who was removed from the analysis) and reaction times (RTs) comprised between 250 and 700 ms for the 4 SIRT conditions. The variability ranges observed for the two behavioral parameters are in agreement with literature and comparable with those reported in other studies (Sternberg, 1966; Cummins and Finnigan, 2007; Tuladhar et al., 2007).

**Table 2:** Mean values of the percentage of correct answers and relative reaction time (RTs) obtained from each participant.

**Subj #**	**Correct answers (%)**				**Reaction time (ms)**			
	**Target**		**NoTarget**		**Target**		**NoTarget**	
	**4 digits**	**6 digits**	**4 digits**	**6 digits**	**4 digits**	**6 digits**	**4 digits**	**6 digits**
1	94	81	94	94	356.91	346.97	422.76	469.35
2	94	78	97	92	373.97	386.68	382.66	444.73
3	100	81	97	86	548.39	468.03	502.80	521.00
4	97	100	94	89	306.37	321.53	305.26	382.34
5	92	72	97	97	411.48	498.77	405.94	511.97
6	94	81	97	89	619.24	632.31	597.43	620.78
7	86	86	94	83	425.81	458.03	483.50	499.70
8	94	86	89	83	473.76	460.26	607.34	554.80
9	83	89	86	78	430.63	509.16	433.10	504.71
10	97	81	92	89	778.11	785.28	704.91	722.66
11	97	97	97	97	319.51	331.20	295.80	371.71
12	97	92	86	94	413.97	347.45	381.87	398.74
13	94	94	92	94	282.38	250.29	265.58	285.88
14	92	81	92	81	444.70	573.41	608.03	595.31
15	97	94	97	89	460.71	474.68	559.97	516.81
16	92	83	94	89	616.64	626.97	580.24	705.78
17	92	83	94	89	616.64	626.97	580.24	705.78
MEAN	94	86	94	89	463.48	476.35	477.50	518.36
STD	4,2	7,6	3,8	5,6	133.01	140.16	128.55	124.12

*Missing answers (RT = 0) were excluded*.

The two-way ANOVA revealed that the percentages of correct answers significantly decreased (94 ± 3 to 87 ± 5%) when the subjects were challenged with the condition of 6 digits with respect to 4 [main factor DIGITS *p* = 0.00007, *F*_(1, 15)_ = 28.15, MSE = 672.8]. The RTs were also significantly longer in the condition 6 digits with respect to 4 digits condition [main factor DIGITS *p* = 0.021, *F*_(1, 15)_ = 6.54, MSE = 13336]. Furthermore, the NoTarget condition was associated to significantly longer RTs as compared to those obtained during the Target condition (470 ± 126 vs. 500 ± 130 ms) in the 6 digits case [main factor TYPE *p* = 0.013, *F*_(1, 15)_ = 7.83, MSE = 12269]. Subject 5 was excluded from the EEG analysis because of low accuracy performance (30% error rate).

### General properties of the networks

The results of the two-way ANOVA for the Local Efficiency (LE), Global efficiency (GE), and Small-Worldness (SW) indices with respect to memory phases and WM load are reported in Table 3 for the five frequency bands.

**Table 3:** Results of two-way repeated measures ANOVA on global indices (*F*-values, ^**^p < 0.001, ^*^p < 0.05).

**Global index**	**Band**	**Phases (d.f. = 2,30)**	**Digits (d.f. = 1,15)**	**Phases × digits (d.f. = 2,30)**
Local efficiency	δ	29.24^**^	4.05^*^	2.81
	θ	27.58^**^	0.02	0.01
	α	30.38^**^	4.66^*^	3.4^*^
	β	79.55^**^	0.11	0.3
	γ	177.77^**^	9.62^*^	8.75^*^
Global efficiency	δ	41.35^**^	5.48^*^	4.68^*^
	θ	21.51^**^	1.2	0.21
	α	39.31^**^	0.03	0.58
	β	10.64^**^	2.98	0.51
	γ	9.19^**^	0.95	1.81
Small-Worldness	δ	57.44^**^	2.09	2.16
	θ	65.32^**^	0.71	1.1
	α	48.84^**^	4.56^*^	4.07^*^
	β	148.16^**^	0.07	0.002
	γ	122.52^**^	0.61	0.51

*FDR correction for multiple ANOVAs was applied. Significant results are highligthed in bold*.

**Figure 2:**
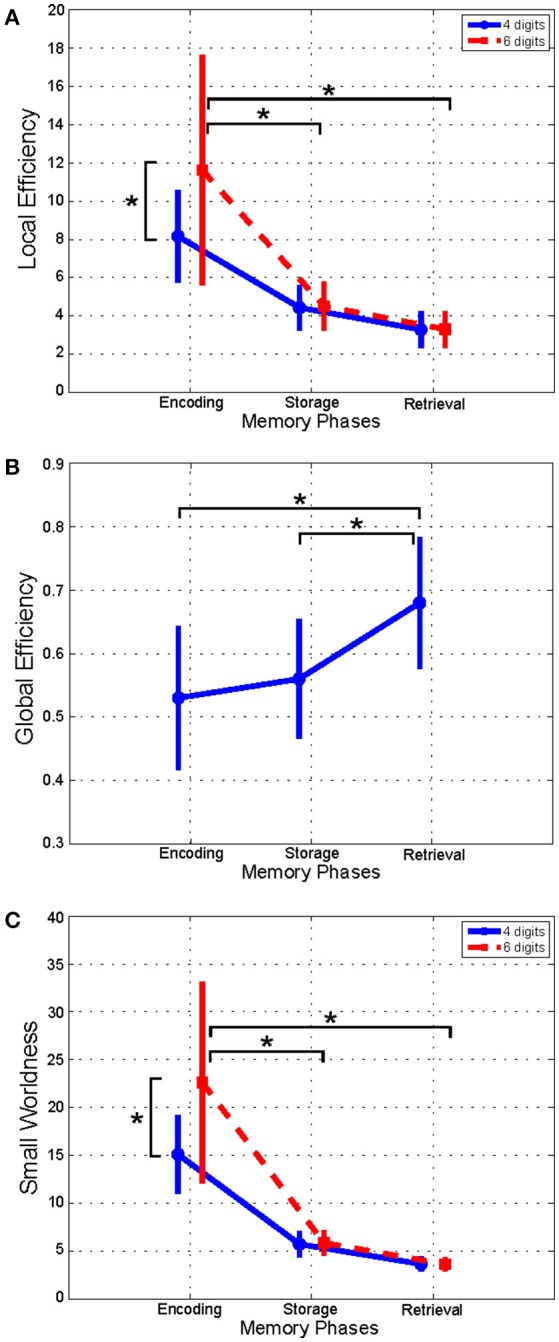
Plot of mean (± *SD*) values of Local Efficiency **(A)**, Global Efficiency **(B)**, and Small-Worldness **(C)** indices estimated in alpha band, and relative to Encoding, Storage and Retrieval phases. The asterisk indicates significant difference (Duncan's *post-hoc*; *p* < 0.05).

As shown in Figure 2, the LE index mean value (*n* = 16) relative to alpha band was significantly higher in the *Encoding* with respect to both *Storage* and *Retrieval* phases (Figure 2A). An opposite trend was observed for the GE index (Figure 2B) that was significantly higher in the *Retrieval* as compared to both *Encoding* and *Storage* (Figure 2B). Finally, the SW index (Figure 2C) was significantly higher in the *Encoding* as compare with *Storage* and *Retrieval*. Similar significant results were found for the three indices in the other frequency bands (Table 3).

We found significant differences between 4 and 6 digits conditions for the LE and the SW indices (Figures 2A,C). In particular, the LE and SW showed significantly higher values for the 6 with respect to 4 digits only during *Encoding* in alpha (Figures 2A,C). Similar results were found in gamma band (Table 3). No significant differences between 4 and 6 digits were found for the GE.

Furthermore, the LE index computed for alpha band and relative to the *Encoding* phase negatively correlated with RTs obtained from both 4 (*r* = −0.7026, *p* = 0.0024) and 6 (*r* = −0.7048, *p* = 0.0023) digits cases.

### Local properties of the networks

**Figure 3:**
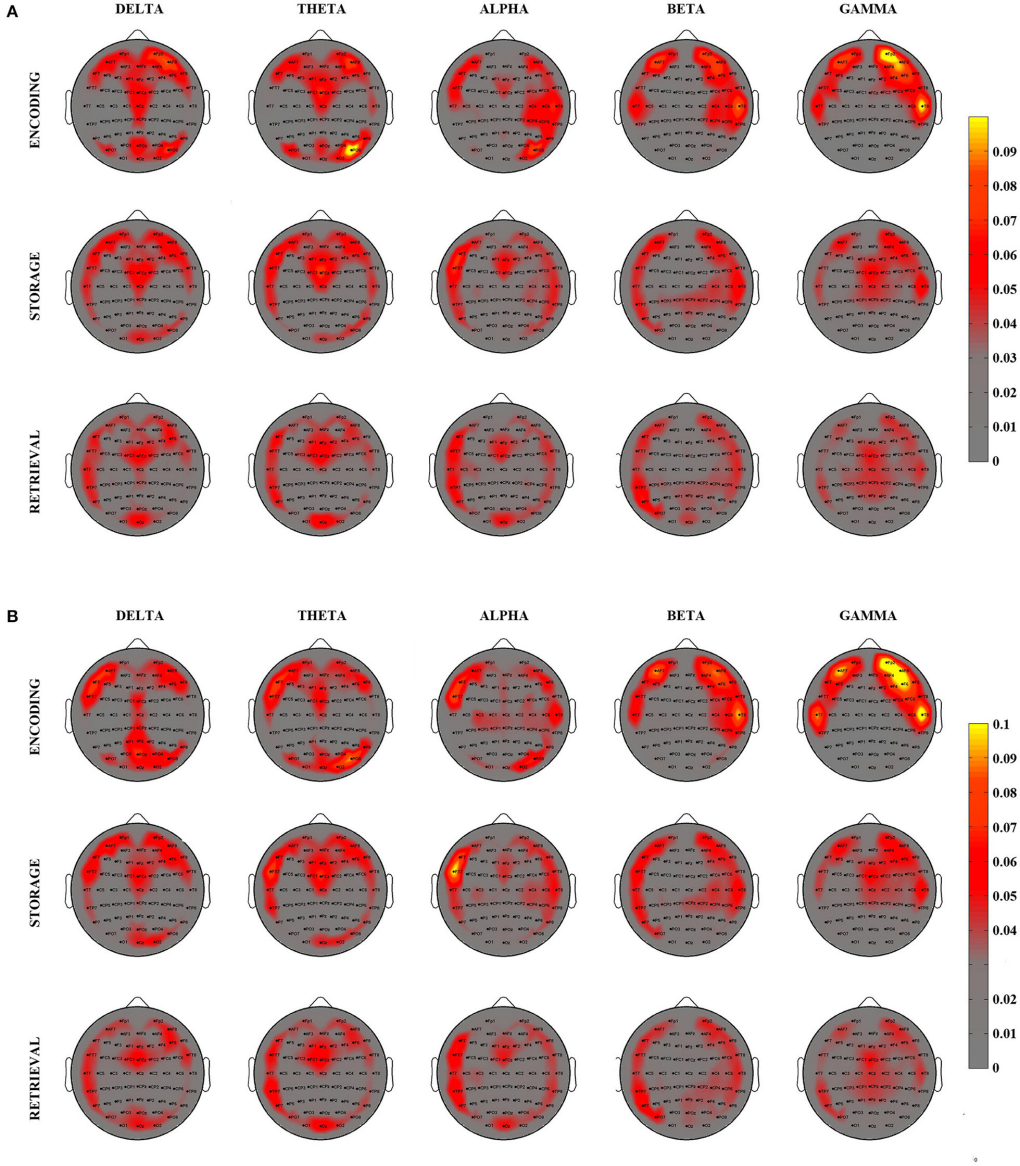
Grand Average Degree–inward and outward—maps relative to the 3 different phases of WM process as elicited by the SIRT (Encoding, Storage, an Retrieval) for 4 digits **(A)** and 6 digits **(B)** conditions and for 5 EEG frequency bands. Degree maps are represented on a 2D scalp model and seen from above. The color of each pixel codes for the corresponding degree magnitude.

The degree index was computed for each electrode and each subject and then averaged within the experimental group for the three PHASES and the two DIGIT conditions [Grand Average (GA) Degree Maps]. A spatial representation of such index is reported in the topographical maps of Figure 3 for 4 digits (Figure 3A) and 6 digits (Figure 3B) cases.

The visual inspection of the GA Degree Maps relative to the 4 digits condition revealed that the three WM phases were associated with distinct connectivity networks for each frequency band oscillation (Figure 3A). During the *Encoding*, we observed a connectivity pattern which mainly included (high degree index) the central midline, the bilateral frontal areas and the bilateral posterior areas in the delta and theta frequency bands. In the alpha band, such patterns were mainly represented over the frontal midline, the left frontal areas and the right hemisphere from frontal to parietal areas. In beta and gamma oscillation ranges, the patterns were prevalent over the bilateral fronto-temporal areas.

*Storage* was consistently associated with a high involvement (high degree) of the bilateral fronto-temporal areas, the frontal midline and the right posterior areas in the delta, theta and alpha bands. Bilateral fronto-temporal areas, left tempo-parietal areas and right central areas have an importal role in the beta band. In gamma band, we found an involvement of bilateral fronto-temporal areas and frontal midline.

The *Retrieval* phase showed a connectivity pattern mainly involving (high degree) frontal-central midline, left fronto-tempo-parietal areas, right frontal areas and occipital areas in the delta, theta, and alpha bands. In beta band, we found a high involvement of bilateral fronto-tempo-parietal areas and parieto-occipital midline. An important role of bilateral fronto-temporal areas and central areas resulted in gamma band.

The averaged patterns obtained for the 6 digits condition are illustrated in Figure 3B. The qualitative (visual inspection) analysis of 6 digits condition revealed a general superimposition with the areas mainly involved in the 4 digits condition.

On the basis of these findings (Figure 3), we selected eight scalp areas (macro-areas) symmetrically distributed over the left and right sides and computed the respective average degree index. The following macro-areas were considered: Left Frontal (Fp1, AF7, F7), Frontal Midline (AFz, Fz, FCz), Right Frontal (Fp2, AF8, F8), Left Temporal (FT7, T7, TP7), Right Temporal (FT8, T8, TP8), Left Parietal (PO7, O1), Right Parietal (PO8, O2), Occipital (Oz).

**Figure 4:**
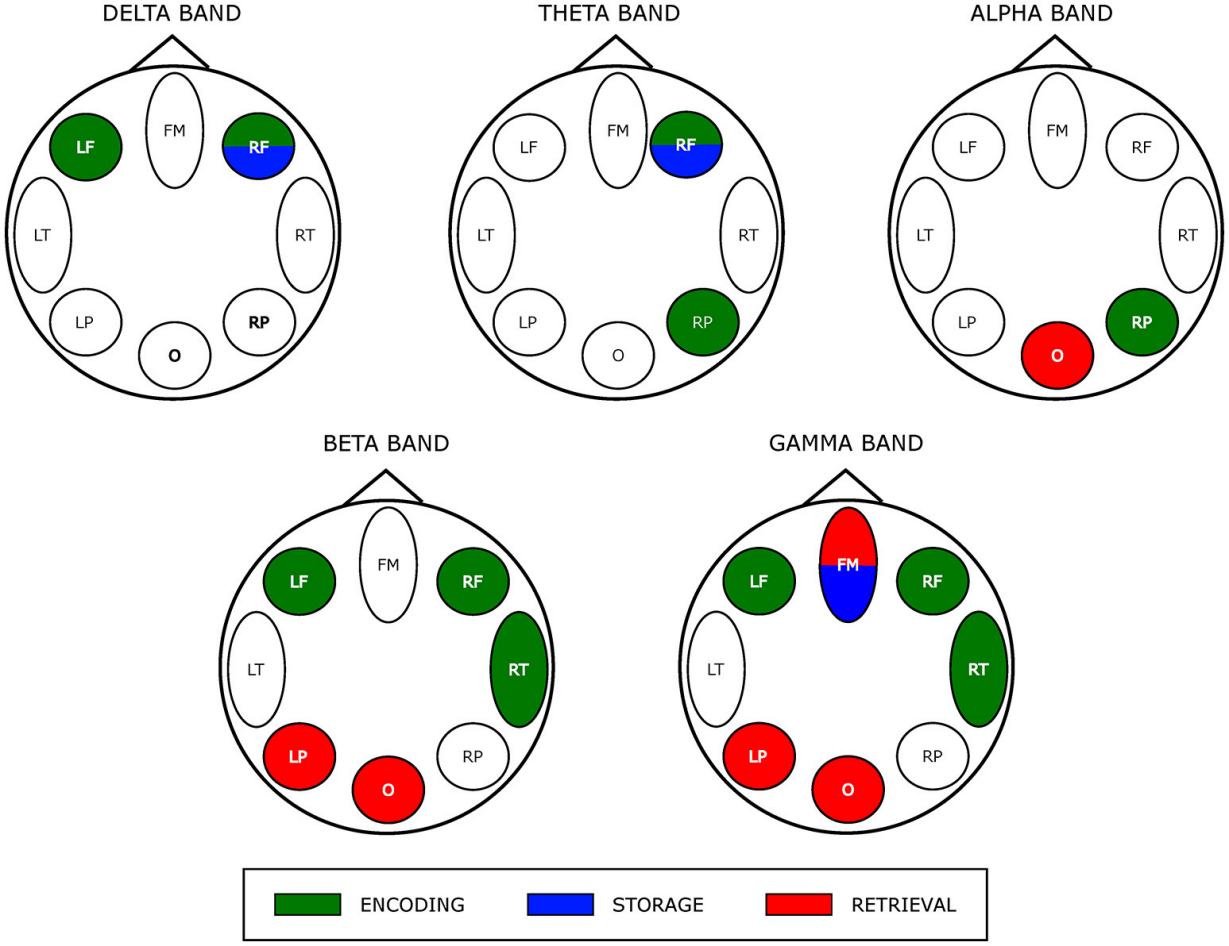
Prevalent network involvement in each WM phase as schematically represented by eight scalp macro-areas for each frequency band. Such schematic representation was derived from the results of the ANOVA obtained for the factor PHASES on macro-areas *Degree* index (see Table 4). We assigned an area to a specific phase if its *Degree* was significantly higher with respect to the other macro-areas.

The results of the two-way ANOVA on degree index with respect to the memory phases and WM load are reported in Table 4 for each macro-area and frequency band. The schematic representation of Figure 4 summarizes the trends obtained for the macro-areas degree across the three memory phases in the five frequency bands (irrespective of DIGIT factor). In particular, the areas distinctive for the Encoding were the bilateral frontal areas in the delta band, the right frontal and right parietal areas in theta band, right parietal area in alpha band, left, and right frontal and right temporal areas in beta and gamma bands. The Storage was instead characterized by right frontal area in both delta and theta bands and frontal midline in gamma band. The retrieval involved occipital area in alpha, left parietal, and occipital areas in beta band and frontal midline, left parietal and occipital areas in gamma band.

**Table 4:** Results of two-way repeated measures ANOVA on local indices (*F*-values, ^**^p < 0.001, ^*^p < 0.01).

**Local index**	**Band**	**Phases (d.f. = 2,30)**	**Digits (d.f. = 1,15)**	**Phases × digits (d.f. = 2,30)**
Left Frontal Degree	δ	4.89^*^	0.34	0.66
	θ	2.67	0.37	2.89
	α	2.12	1.31	1.65
	β	4.11^*^	0.38	0.14
	γ	26.16^**^	4.77	1.48
Frontal Midline Degree	δ	0.13	1.34	1.06
	θ	1.42	0.72	0.38
	α	0.11	0.65	0.65
	β	2.31	0.49	0.05
	γ	6.39^*^	0.01	0.83
Right Frontal Degree	δ	12.81^**^	3.05	6.92^*^
	θ	4.98^*^	0.12	1.05
	α	0.58	0.13	0.08
	β	12.16^**^	2.23	0.74
	γ	20.56^**^	6.51^*^	3.22
Left Temporal Degree	δ	0.66	4.49	0.83
	θ	3.01	2.33	0.46
	α	1.39	7.62^*^	2.84
	β	0.78	2.02	0.57
	γ	0.53	0.001	0.75
Right Temporal Degree	δ	1.72	0.38	1.49
	θ	1.5	0.19	0.73
	α	1.53	0.34	0.93
	β	9.14^**^	0.38	2.52
	γ	15.35^**^	0.09	0.27
Left Parietal Degree	δ	0.64	0.9	0.68
	θ	1.11	0.12	1.55
	α	0.46	0.93	0.42
	β	7.55^*^	1.15	0.79
	γ	26.74^**^	4.89^*^	2.08
Occipital Degree	δ	0.45	0.2	0.71
	θ	1.22	0.22	1.55
	α	5.63^*^	1.16	1.04
	β	8.95^**^	0.43	2.49
	γ	6.42^*^	0.49	5.04^*^
Right Parietal Degree	δ	**2.87**	0.96	0.59
	θ	11.19^**^	0.09	0.09
	α	5.75^*^	0.009	0.14
	β	0.17	0.01	0.2
	γ	0.14	0.001	0.17

*FDR correction for multiple ANOVAs was applied. Significant results are highligthed in bold*.

## Discussion

This study applied a graph theory—driven approach to complex causality patterns derived from EEG recordings with the aim to identify distinct topological properties of the neural networks associated to encoding, storage, retrieval phases of WM as elicited during visual SIRT performed by healthy, middle age adults. We found that, during the encoding phase, the global network exhibited a small-world topology (in all frequency bands) thus, indicating a network configuration accounting for both global information transfer and local processing. The requirement of such *optimal* configuration specifically for item encoding appeared further corroborated by the negative correlation between local efficiency and behavioral task performance. The small-world configuration of the whole network persisted across maintenance and rehearsal of encoded items but it showed a descendent trend. At the local scale, the degree of information flow between scalp regions was selective for each of the three different WM phases, in that *network hubs* were consistent with the different role of brain regions in different WM phases. Overall, these EEG findings provide evidence that the complexity of functional networks underpinning the model of multicomponent WM (Baddeley, 2012; Eriksson et al., 2015) may be represented by synthetic EEG indices which preserve the selectivity of the dynamics occurring during encoding, maintenance and rehearsal of memory items.

### Behavioral results

The behavioral results obtained from our sample of healthy, middle age adults are in line with what was reported by previous studies conducted in healthy adults and where the SIRT was applied to investigate working memory (WM) processes (Sternberg, 1966; Cummins and Finnigan, 2007; Tuladhar et al., 2007). As expected, WM loads (4, 6 digits conditions) had a significant effect on the response time and accuracy for both Target and NoTarget probes in our sampled population. These WM load-related behavioral changes have been previously ascribed to a serial scanning of memorized elements required in order to recall the memorized material (Majerus et al., 2006).

### Global organization of the WM network

The complex human brain networks have been found to have a “small-world” topology which is characterized by a high local specialization and a high global integration to sustain a high efficiency at a low wiring cost (Sporns, 2013a).

The significant effect of phase factor on all the global indices (global and local efficiency, small-worldness; Table 3) indicated that a small-word topology of the networks was present in all three WM phases with the highest value associated with encoding with respect to storage and retrieval (Figure 2; in alpha band). Such descendent trend was evident for lower to higher frequency oscillations (Table 3).

The finding of a descendent trend in the network small-worldness in all frequency bands may reflect a general network tendency to reduce local segregation (see also local efficiency decrease) in favor of global integration (see global efficiency increase) of the information exchange between/within the different brain regions as cognitive processing evolves from encoding to retrieval. Such dynamics in the topological re-arrangement is consistent with the recently released global workspace theory (Baars and Franklin, 2003; Baars et al., 2013) postulating that the networks structure reorganizes across the temporal evolution of WM cognitive processing (Bola and Sabel, 2015). As such, the dynamics observed in the EEG-derived network(s) would reinforce the assumption at the base of multi-store model revised by Baddeley (2012) that the several cognitive components (i.e., central executive, episodic buffer (s), phonological loop and visuo-spatial sketchpad) are not “crystallized” but have a tendency to be “fluid” as well the capacity they sub-serve (attention, temporary storage…; Baddeley, 2012). In particular, this dynamic observed for the topology networks might reflect the operational mode of the “episodic buffer” component of the Baddeley model (Baddeley, 2010). This “buffer” serves as an intermediary between the storage subsystems with different codes (i.e., phonological loop and visuo-spatial sketchpad) whose content is bounded by the buffer into unitary multi-dimensional representations. Thus, one can speculate that a tendency toward a more global vs. local integration network topology (ie, the decrease of small-worldness across WM phases) would “*optimally*” serve the function of the episodic buffer by favoring the information flow between WM networks (i.e., 2 storage subsystems).

The dynamic changings toward a more globally integrated network(s) interplay with less specialized segregation could also be effective in sustaining more recent WM models (for review see Eriksson et al., 2015). In this recent reappraisal of WM functioning, the content of working memory would be defined by an interaction between selective perceptual (visual, auditory…) information process (operated via a selective attention) and LTM representations being in a particular state of “accessibility” that requires a largely persistent activity of specialized networks controlled by attentional processes. Thus, a whole brain network with high global information transfer (integration) would better “sustain” an optimal interplay between locally specialized networks (see below, local organization of WM subnetworks).

The encoding process directly influences the precision and accuracy of subsequent WM representations (Awh and Vogel, 2008; Rutman et al., 2010). The well-known limitation in the capacity to simultaneously encode objects requires efficient mechanisms to operate a selection of only the most relevant objects from the immediate environment to be represented in WM by restricting those irrelevant from consuming capacity (Vogel et al., 2005; Chun, 2011). In this regard, several evidence indicate that a successful encoding information into WM is the result of an interplay between brain circuits underlying selective attention processes and perceptual (e.g., visual) object representation (for a review Gazzaley and Nobre, 2012) that, in a more recent vision would trigger LTM object representation (Eriksson et al., 2015). A small-world topology could well account for this complex network interplay by supporting both specialized and integrated information processing in the whole brain connectivity. It comprises both high segregated (or modular) processing (high clustering) and distributed (or integrated) processing (short path length; Bassett and Bullmore, 2006). The observed high small-world network during WM encoding (with respect to maintenance and retrieval) would fit with the necessity of the brain to combine the functioning of specialized (segregated) modules with a number of inter-modular links integrating those modules.

To further corroborate this interpretation, it is notable to mention that a disruption of an *optimal* small-world network organization has been described in schizophrenic patients (Fornito et al., 2012) who exhibited an impairment of WM performance accounted by a decreased efficiency in item encoding (Cairo et al., 2006; Koch et al., 2009). As yet, altered oscillatory dynamics during encoding of information have been reported in normal and pathological aging associated with cognitive decline (Aine et al., 2011; Kirova et al., 2015; Proskovec et al., 2016).

We also found that the network *optimal* topology defined by a high local efficiency and small-worldness, increased significantly as a function of WM load increase (4 vs. 6 digits) only during encoding. This WM load-induced modulation of network topology reinforces the above interpretation of a high network modularity required during encoding. Recent neurophysiological evidences support the idea that visual WM capacity limitation (i.e., the so-called *set size effect* Luck and Vogel, 1997) begins with neural attention resource allocation at encoding (Gurariy et al., 2016). Of note, the WM load-induced increase in the values of the network global indices was selectively observed in the alpha and gamma frequency bands. Alpha oscillations have been hypothesized to play an active role in protecting WM items from non-relevant information (Jensen and Mazaheri, 2010) by suppressing distracting sensory information (Romei et al., 2010). More specifically, the increase of WM load is associated to an increase of alpha-band coherence between midline parietal and left temporal/parietal sites during encoding (Payne and Kounios, 2009). From the behavioral view point, we found that the network local efficiency estimated in alpha band and relative to encoding varied as function of the RTs (negative correlation; Figure 3). The existence of such correlation exclusively in alpha band is in accordance with previous evidence of a correlation between changes in α-power spectrum and behavioral performance during encoding (Klimesch, 1999; Bashivan et al., 2014). The increase in the values of the indices describing the global network organization during encoding was also selective for the gamma frequency. As such this finding is in line with evidence of a direct correlation between the changes in the gamma power spectrum amplitude and the number of items to be memorized (Howard et al., 2003; Roux et al., 2012; Roux and Uhlhaas, 2014).

### Local organization of the working memory networks

The computation of local degree maps (GA illustrated in Figure 3) allowed for the identification of *hubs* within the WM network(s) activated across the three phases elicited by the visual SIRT. As expected, encoding, storage and retrieval WM phases were consistently characterized by a main involvement of bilateral *frontal* and *temporal* regions in all frequency oscillations while an *anterior-to-posterior midline* pattern was prevalent in the low (delta/theta) EEG frequency range. In addition, a bilateral *parieto-occipital connectivity* was observed mainly in theta/delta oscillations during the encoding, while storage/retrieval phase were characterized by a prevalent *left temporo-parietal and right fronto-parietal connectivity* in alpha/beta bands. These patterns were sensitive to WM load increase.

Consistently with the view of WM as emerging from the dynamic interplay of several brain regions, recent evidences indicate that the integrity of white matter pathways connecting the dorsolateral frontal cortex, parietal cortex, and temporal cortex correlates with working-memory performance (Charlton et al., 2010). Within this large network of areas, the prefrontal cortex has been suggested to be crucial for executive demands such as the maintenance of resilient information during WM, updating WM content, and shifting (Nee et al., 2013). Together with the prefrontal areas, the parietal cortex is also causally involved in WM functioning, being associated with executive aspects (superior parietal cortex) of WM (Collette et al., 2005) and the implementation of selective attentional control (Awh et al., 2006). Interestingly, parietal cortex activity correlates with WM capacity in that its activity increases as the number of items to remember increases (Vogel and Machizawa, 2004). According to computational modeling (O'Reilly, 2006), the basal ganglia (striatum) would play a key role in controlling (filtering) when the prefrontal cortex representations should be maintained vs. updated. Moreover, the above mentioned parietal load effect negatively correlated with basal ganglia activity (McNab and Klingberg, 2008).

Our connectivity patterns expressed as locally distributed *hubs* of information flow between scalp regions (i.e., local degree index) well reflect the main interpretational mapping of WM processes to brain regions, thus highlighting the accuracy of our EEG network estimation approach in providing indices which can specifically describe the distributed topography of the networks involved in WM task solving. The spectral features of the estimated local topological indices further corroborate their selectivity in capturing the (local) functional dynamics underlying WM processing.

There are a number of evidence that an interplay between rhythmic activity at low (delta/theta) and high (beta/gamma) frequency has been suggested to enable WM item encoding and maintenance in humans (for review see Roux and Uhlhaas, 2014). Particularly, the gamma band would be relevant for active maintenance of WM information, whereas theta band would be involved in the temporal organization of WM items. The relevance of alpha oscillation would reside in filtering task non-relevant information.

As schematically illustrated in Figure 4, we actually found that *Encoding* networks were mainly described by *hubs* (encoding—related local degree indices contrasted against those of storage and retrieval time series) located within bilateral frontal and right fronto-temporal scalp area in low (delta/theta) and in the high frequency oscillation range (beta/gamma), respectively.

As discussed above, gamma band activity plays a role in maintenance of visual (and others sensory) WM items (Tallon-Baudry et al., 1998; Kaiser et al., 2008) which spatially occurs within the prefrontal cortex in association with parietal cortex (Eriksson et al., 2015). In addition, EEG/MEG source localization studies pointed out that gamma oscillatory activity changes (increased power) is mainly localized over frontal (and parietal) regions (Palva and Palva, 2012). The exclusive bilateral frontal areas involvement for delta activity-related hubs would be consistent with the role of sustained delta activity in inhibiting interferences that might affect task performance (Harmony, 2013; Kleen et al., 2016).

Finally, the observed lateralization of network hubs toward right parietal area in alpha/theta during encoding might be related to spatial WM (Owen et al., 2005) elicited by a visual SIRT. Contemporary views of alpha/theta range of frequency suggest that it reflects the allocation of spatial attention to the memoranda, as well as the suppression of distracting information (Corbetta and Shulman, 2002; Asplund et al., 2010; Klimesch, 2012).

The *storage* partially overlaps *encoding* network hub map as it involved (right) frontal area in delta/theta frequency range. Although the correspondence in neural activity between encoding and maintenance still remains debatable (Gazzaley et al., 2004; Woodward et al., 2006; Chang et al., 2007) recent work by Cohen et al. (2014) provides empirical evidence for an overlap between encoding and maintenance processes as a critical element of WM (Postle, 2006; D'Esposito, 2007). A gamma-related frontal midline hub also was observed (in common with *retrieval*) that also could reflect the interplay between subcortical structures (i.e., basal ganglia) and (pre) frontal areas cortex responsible for maintenance of relevant WM items (Kaiser et al., 2009; Roux et al., 2012).

During *retrieval* we observed a selective distribution of network hubs within occipital area in alpha, beta and gamma bands as well as within the left parietal region in high frequency oscillations (beta/gamma band). Such parieto-occipital engagement could account for visual stimulus presentation and visual information processing during retrieval (Voytek et al., 2010). Moreover, neuronal synchronization in the gamma band over occipital areas has been associated to subject ability during encoding and retrieval memory phases (Osipova et al., 2006).

As expected local degree indices varied as function of WM load. Specifically, the left temporal degree increased as function of WM load in alpha band. This finding is consistent with a role of (left) temporal region in sub-lexical phonological processing of visual material (Howard et al., 1992; Price, 1998). During Sternberg tasks sequential encoding would activate the phonological loop to support the maintenance of sequenced WM items by means of subvocal rehearsal (silent speech; Henson et al., 2000; Barry et al., 2011). We found that frontal and parietal local degree indices were sensitive to WM load in gamma oscillations that well reflect the direct correlation between gamma power magnitude and the number of items to be memorized and the role of sustained activity in frontal and parietal areas for maintenance of memoranda (Howard et al., 2003; Roux et al., 2012; Roux and Uhlhaas, 2014).

The present EEG-derived network findings still await for further consolidation which requires a large sample of different age population to be involved and a network model testing procedure such as challenging the distributed network hub mapping both internally (e.g., leave-one-out approach to verify the fault tolerance of the network to the removal of one node) and/or externally (e.g., with non-invasive technique to induced virtual lesions such as rTMS).

Upon consolidation, our EEG-derived network estimation approach may on the one hand, break new ground in the WM function theoretical modeling and on the other it would offer a valuable and affordable method to improve clinical assessment and evaluate treatment efficacy of cognitive disorders occurring after brain lesions (i.e., stroke).

## Author contributions

JT: EEG experimental data analysis management; manuscript writing; LA: experimental design definition, EEG derived brain network data analysis supervision and validation; MR: interpretation of behavioral data; AA: implementation of methodological pipeline (connectivity estimation and graph theory approach); SK: EEG and behavioral data collection; GW: experimental design definition and supervision of data collection; DM: responsible for study; study design and management; overall data interpretation; manuscript writing management.

### Conflict of interest statement

The authors declare that the research was conducted in the absence of any commercial or financial relationships that could be construed as a potential conflict of interest. The reviewer BM and handling Editor declared their shared affiliation, and the handling Editor states that the process nevertheless met the standards of a fair and objective review.
